# Systematisches Review zur Schätzung der Prävalenz entzündlich rheumatischer Erkrankungen in Deutschland

**DOI:** 10.1007/s00393-022-01305-2

**Published:** 2023-01-02

**Authors:** Katinka Albrecht, Sebastian Binder, Kirsten Minden, Denis Poddubnyy, Anne C. Regierer, Anja Strangfeld, Johanna Callhoff

**Affiliations:** 1 Programmbereich Epidemiologie und Versorgungsforschung, Deutsches Rheuma-Forschungszentrum Berlin, Charitéplatz 1, 10117 Berlin, Deutschland; 2 Klinik für Pädiatrie mit SP Pneumologie, Immunologie und Intensivmedizin, Charité Universitätsmedizin Berlin, Berlin, Deutschland; 3 Rheumatologie am Campus Benjamin Franklin – Medizinische Klinik für Gastroenterologie, Infektiologie und Rheumatologie, Charité Universitätsmedizin Berlin, Berlin, Deutschland; 4 Medizinische Klinik mit Schwerpunkt Rheumatologie und Klinische Immunologie, Charité Universitätsmedizin Berlin, Berlin, Deutschland; 5 Institut für Sozialmedizin, Epidemiologie und Gesundheitsökonomie, Charité Universitätsmedizin Berlin, Berlin, Deutschland

**Keywords:** Rheumatoide Arthritis, Spondyloarthritis, Juvenile idiopathische Arthritis, Kollagenosen, Vaskulitis, Rheumatoid arthritis, Spondylarthritis, Juvenile idiopathic arthritis, Connective tissue diseases, Vasculitis

## Abstract

**Zielsetzung:**

Es erfolgt eine aktualisierte Schätzung der Prävalenz entzündlich rheumatischer Erkrankungen (ERE) in Deutschland.

**Methodik:**

Mit einer systematischen Literaturrecherche in PubMed und Web of Science (letzte Suche am 08.11.2022) wurden Originalartikel (regionale und bundesweite Surveys und Routinedatenanalysen für Arthritiden, Kollagenosen und Vaskulitiden) zur Prävalenz von ERE für den Zeitraum 2014 bis 2022 identifiziert. Datenquellen, Erhebungszeitraum, Diagnosedefinition und das Risiko einer Verzerrung werden berichtet. Die Prävalenzen wurden anhand der verfügbaren Daten unter Berücksichtigung internationaler Angaben geschätzt.

**Ergebnisse:**

Die Suche durch 2 Autorinnen ergab 263 Treffer, von denen 18 Routinedatenanalysen und 2 Surveys die Einschlusskriterien erfüllten. Die Prävalenzangaben lagen bei 0,42–1,85 % (rheumatoide Arthritis), 0,32–0,5 % (ankylosierende Spondylitis), 0,11–0,32 % (Psoriasisarthritis), 0,037–0,14 % (systemischer Lupus erythematodes), 0,07–0,77 % (Sjögren/Sicca-Syndrom), 0,14–0,15 % (Polymyalgia rheumatica, ab 40 Jahre), 0,04–0,05 % (Riesenzellarteriitis, ab 50 Jahre) und 0,015–0,026 % (ANCA-assoziierte Vaskulitis). Das Bias-Risiko war in 13 Studien moderat, in 7 Studien hoch. Anhand dieser Ergebnisse schätzen wir die Prävalenz von ERE in Deutschland auf 2,2–3,0 %. Dies entspricht in etwa 1,5 bis 2,1 Mio. Betroffenen. Die Prävalenz der juvenilen idiopathischen Arthritis wurde mit ca. 0,10 % (0,07–0,13 %) der 0‑ bis 18-Jährigen angegeben, was etwa 14.000 Kindern und Jugendlichen in Deutschland entspricht.

**Schlussfolgerung:**

Dieses systematische Review zeigt einen Anstieg der Prävalenzen von ERE in Deutschland, basiert jedoch fast ausschließlich auf Routinedatenanalysen. In Ermangelung mehrstufiger Bevölkerungsstudien sind die vorliegenden Daten bei moderat bis hohem Verzerrungsrisiko insgesamt unsichere Quellen für Prävalenzschätzungen.

**Zusatzmaterial online:**

Zusätzliche Informationen sind in der Online-Version dieses Artikels (10.1007/s00393-022-01305-2) enthalten.

## Abstract (en)

**Objective:**

To update the estimated prevalence of inflammatory rheumatic diseases (IRD) in Germany.

**Methods:**

A systematic literature search in PubMed and Web of Science (last search 8 November 2022) identified original articles (regional and nationwide surveys and routine data analyses for arthritides, connective tissue diseases, and vasculitides) on the prevalence for the period 2014–2022. Data sources, collection period, case definition, and risk of bias are reported. The prevalences were estimated from available national data, with consideration of international data.

**Results:**

Screening by 2 authors yielded 263 hits, of which 18 routine data analyses and 2 surveys met the inclusion criteria. Prevalence data ranged from 0.42% to 1.85% (rheumatoid arthritis), 0.32–0.5% (ankylosing spondylitis), 0.11–0.32% (psoriatic arthritis), 0.037–0.14% (systemic lupus erythematosus), 0.07–0.77% (Sjoegren’s disease/sicca syndrome), 0.14–0.15% (polymyalgia rheumatica, ≥ 40 years), 0.04–0.05% (giant cell arteritis, ≥ 50 years), and 0.015–0.026% (ANCA-associated vasculitis). The risk of bias was moderate in 13 and high in 7 studies. Based on the results, we estimate the prevalence of IRD in Germany to be 2.2–3.0%, which corresponds to approximately 1.5–2.1 million affected individuals. Prevalence data of juvenile idiopathic arthritis was reported to be around 0.10% (0.07–0.10%) of patients 0–18 years old, corresponding to about 14,000 children and adolescents in Germany.

**Conclusion:**

This systematic review shows an increase in the prevalence of IRD in Germany, which is almost exclusively based on routine data analyses. In the absence of multistage population studies, the available data are overall uncertain sources for prevalence estimates at moderate to high risk of bias.

Für die Versorgungsbedarfsplanung in der Rheumatologie werden aktuelle Zahlen zur Häufigkeit entzündlich rheumatischer Erkrankungen in Deutschland benötigt. Neben tatsächlichen Veränderungen in der Häufigkeit der Erkrankungen können die Alterung der deutschen Bevölkerung, aber auch die verbesserte Frühdiagnostik und ein Rückgang in der Mortalität zu einem Anstieg der Prävalenzen in den letzten Jahren geführt haben. Da es in Deutschland kein Bevölkerungsregister für entzündlich rheumatische Erkrankungen gibt, kann die Prävalenz nur anhand verfügbarer nationaler Daten und ergänzt um europäische Daten annäherungsweise geschätzt werden. Im Jahr 2016 haben wir die Häufigkeit und Verteilung von Krankheiten der Bewegungsorgane anhand der verfügbaren Evidenz berechnet [1]. Ausgehend von diesen Daten, soll in dieser Arbeit neu verfügbare Evidenz bis 2022 mit einem systematischen Review ermittelt und es sollen die Häufigkeiten an die aktuellen Bevölkerungszahlen angepasst werden.

## Methodik

Es wurde eine systematische Literaturrecherche (SLR) in den Datenbanken PubMed und Web of Science für den Zeitraum vom 01/2014 (Ende der bisherigen Referenz [1]) bis 10/2022 durchgeführt (letzte Suche am 08.11.2022). Eingeschlossen wurden Originalarbeiten in deutscher oder englischer Sprache, die die Prävalenz entzündlich rheumatischer Erkrankungen (Arthritiden, Kollagenosen, Vaskulitiden) in Deutschland bei Erwachsenen oder Kindern und Jugendlichen untersucht haben. Folgende Suchterms wurden verwendet: prevalence, rheumatoid arthritis, spondyloarthritis, spondylitis, lupus erythematodes, polymyalgia rheumatica, sjoegren’s disease, inflammatory myositis, ANCA-associated vasculitis, rheumatic disease. Die Suchstrategie und der Suchprozess sind im Zusatzmaterial online dargestellt. Die Literaturauswahl erfolgte durch 2 Personen (KA, JC) unabhängig voneinander, bei Unstimmigkeiten wurde ein Konsens herbeigeführt. Ergänzend erfolgte eine Suche in den Referenzen der ausgewählten Publikationen, durch Befragung von Experten nach weiteren Studien und durch Internetrecherche. Von 263 Artikeln erfüllten 16 die Einschlusskriterien, und 4 Artikel wurden durch manuelle Suche ergänzt. Eingeschlossene Studien wurden folgenden Krankheitsbildern zugeordnet: rheumatoide Arthritis (RA), axiale Spondyloarthritis (axSpA) bzw. ankylosierende Spondylitis (AS), Psoriasisarthritis (PsA), systemischer Lupus erythematodes (SLE), Sjögren, Polymyalgia rheumatica (PMR), Riesenzellarteriitis (RZA), ANCA-assoziierte Vaskulitiden und juvenile idiopathische Arthritis (JIA).

Die eingeschlossenen Artikel wurden anhand einer Checkliste, adaptiert nach Hoy et al. [2], auf das Risiko einer Verzerrung („risk of bias“) überprüft. Für jede Studie wurden 4 Aspekte der externen und 5 Aspekte der internen Validität überprüft und jeweils zweistufig bewertet (hohes bzw. geringes Verzerrungsrisiko). Im aus diesen Bewertungen gebildeten Gesamturteil wurde das Risiko einer Verzerrung als hoch, moderat oder niedrig eingestuft. Eine Metaanalyse auf Basis dieser SLR wurde bewusst nicht durchgeführt, da die gefundenen Arbeiten in vielen Kriterien nicht vergleichbar sind und verschiedene methodische Voraussetzungen für eine Metaanalyse nicht erfüllt sind. Für die Einordnung der Daten aus Deutschland und die daraus abgeleiteten Schätzungen haben wir zusätzlich europäische Literatur, systematische Reviews und deutsche Übersichtsarbeiten herangezogen, diese sind aber nicht Bestandteil der SLR. Die Arbeit wurde in Übereinstimmung mit den PRISMA reporting guidelines verfasst [3].

## Ergebnisse

Anhand der SLR wurden 18 Routinedatenanalysen und 2 Surveys identifiziert. Die Prävalenzangaben und die jeweiligen Datenquellen sind in Tab. 1 berichtet.

**Table 1:** 

Referenz	Diagnose	Datenquelle	Studienpopulation	Diagnose Definition	Untersuchungszeitraum	Prävalenz Rohdaten	Prävalenz bei Frauen/Männern	Standardisierung
Schmidt 2020 [4]	RA AS SLE Sjögren	Bundesweite NAKO-Gesundheitsstudie	101.779 Befragte (20 bis 69 Jahre)	Patientenberichtete ärztliche Diagnose (jemals)	2014–2017 (Prävalenz geschätzt aus alters- und geschlechtsstratifizierter Zufallsstichprobe; einmalige Befragung im Zeitraum)	*RA*: 1,85 % *AS*: 0,49 % *SLE*: 0,14 % *Sjögren*: 0,07 %	RA: 2,62 %/1,08 % AS: 0,42 %/0,55 % SLE: 0,23 %/0,05 % Sjögren: 0,13 %/0,01 %	Alters- und geschlechtsstandardisiert auf deutsche Standardbevölkerung 2011
Kienitz 2020 [5]	RA	Bundesweite GKV-Routinedaten	Ca. 2,3 Mio. Versicherte ≥ 18 Jahre	(1) ICD: M05, M06; (2) Facharztdiagnose (3) ICD-code + DMARD	2008–2013 2013 (Jährliche Prävalenz im jeweiligen Jahresquerschnitt)	(1) 2008–2013: 1,17–1,34 % (2) 2011–2013: 0,94–1,07 % (3) 2008–2013: 0,44–0,54 %	(1) 1,8 %/0,8 % (2013)	Keine Standardisierung
Grellmann 2020 [6]	RA PsA	Bundesweite GKV-Routinedaten	965.759–1.930.158 (unterschiedlich in den betrachteten Jahren) ≥ 18 Jahre	RA: M05.8, M06.0, M06.8 PsA: M07.0–3, L40.5	2012–2016 (Jährliche Prävalenz im jeweiligen Jahresquerschnitt)	*RA*: 0,42–0,53 % (2012–2016) *PsA*: 0,27–0,32 %	Frauen im gebärfähigen Alter: RA: 0,2 % PsA: 0,1–0,2 %	Alters- und geschlechtsstandardisiert auf GKV-Gesamtpopulation im jeweiligen Jahr
Strahl 2018 [7]	RA	Regionale AOK-Routinedaten	3.446.670 Versicherte	(1) ICD: M05, M06 (2) + Medikation	2013	(1) 1,05 % (2) 0,64 %	(1): 1,4 %/0,64 % (2) 0,86 %/0,39 %	Altersstandardisiert auf „alte“ Europastandardbevölkerung von 1976
Steffen 2017 [8]	RA	Bundesweite GKV-Routinedaten	60–61 Mio. Versicherte	(1) M05, M06 + Labor (2) 2014: mindestens 1‑mal ICD-Codes + Labor im Gesamtzeitraum	2009–2015 (jährliche Prävalenz im jeweiligen Jahresquerschnitt)	(1) 2009: 0,87 % (0,87 %) (1) 2015: 1,08 % (1,06 %) (2) 2014: 1,23 % (1,20 %)	1,49 %/0,62 % (2015)	Alters- und geschlechtsstandardisiert auf GKV-Gesamtpopulation 2016
Hense 2016 [9]	RA	Bundesweite BARMER-Routinedaten	7.155.315 Versicherte	(1) M05, M06 (2) + Labor (3) + Medikation (4) + Rheumatologie	2013 (Jahresprävalenz)	(1) 1,62 % (1,38 %) (2) 1,11 % (0,95 %) (3) 0,94 % (0,81 %) (4) 0,64 % (0,55 %)	–	Alters- und geschlechtsstandardisiert auf deutsche Standardbevölkerung 2013
Krüger 2018 [10]	AS	Bundesweite GKV-Routinedaten (InGef)	3,2 Mio. Versicherte	M45	2013 (Jahresprävalenz)	322/100.000	–	Datenbank repräsentativ für deutsche Bevölkerung nach Geschlecht und Alter, deshalb keine gesonderte Standardisierung
Deike 2021 [11], Sewerin 2019 [12]	PsA	Bundesweite GKV-Routinedaten	64–65 Mio. Versicherte	Keine Angabe	2009–2012 (jährliche Prävalenz im jeweiligen Jahresquerschnitt)	2009: 0,20 % 2012: 0,24 %	0,21–0,25 %/0,18–0,21 %	Keine Standardisierung für Gesamtschätzer
Reinhardt 2021 [13]	PsA, juvenile PsA	DAK-Routinedaten	2,319,584 Versicherte	M07.0–3, M09.0 (juvenil)	2010 (Jahresprävalenz)	0,31 % (0,29 %) Juvenil: 0,01 % (0,01 %)	–	Alters- und geschlechtsstandardisiert auf GKV-Gesamtpopulation 2012
Sondermann 2018 [14]	PsA	Regionale AOK-Routinedaten	Ca. 2,8 Mio. Versicherte	L40.5	2014 (Quartal 1 und 2)	0,11 %	–	Keine Standardisierung
Rech 2020 [15]	PsA	Bundesweite Routinedaten (InGef)	2,9 Mio. erwachsene Versicherte	M07.0, M07.1, M07.3	2012–2017 (kumuliert)	2017: 0,15 %	–	Datenbank als repräsentativ für deutsche Bevölkerung nach Geschlecht und Alter angenommen, deshalb keine gesonderte Standardisierung
Schwarting 2021 [16]	SLE	Bundesweite BKK-Routinedaten	4,1 Mio. erwachsene Versicherte	M32.1,8,9 + Labor/Medikation/Facharztdiagnose	2009–2014 (jährliche Prävalenz im jeweiligen Jahresquerschnitt)	2009: 37,3 (38,6)/100.000 2014: 47,4 (48,5)/100.000 Mit statistischer Adjustierung wegen rechtszensierter Daten in 2014: 55,8/100.000	2014: 79,8/13,8 pro 100.000	–
Brinks 2014 [17]	SLE	Bundesweite GKV-Routinedaten	2,3 Mio. Versicherte	M32	2002 (Jahresprävalenz)	36,7 (34,3–39,3)/100.000	55,4/15,4 pro 100.000	Keine Standardisierung
Albrecht 2020 [18]	Sjögren	Bundesweite BARMER-Routinedaten	7,2 Mio. Versicherte ≥ 18 Jahre	M35.0	2007–2018 (jährliche Prävalenz im jeweiligen Jahresquerschnitt)	2007–2018: 0,68–0,77 %	0,87–0,97 %/0,38–0,44 % pro 100.000	Keine Standardisierung
Colombo 2022 [19]	PMR	Regionale AOK-Routinedaten	Keine Angabe ≥ 40 Jahre	M35.3, M31.5	2011–2019 (jährliche Prävalenz im jeweiligen Jahresquerschnitt und kumuliert)	2011: 115(107)/100.000 2019: 153(145)/100.000 Kumuliert: 139(130)/100.000	166/86 pro 100.000 (Kumuliert, altersstandardisiert)	Alters- und geschlechtsstandardisiert auf GKV-Gesamtpopulation 2019
Herlyn 2014 [20]	RZA AAV	Regionales Survey	469.000 Einwohner	RZA: M31.5, M31.6 GPA: M31.3, EGPA: M30.3, MPA: M31.7 + CHCC Definition, ACR-Kriterien	2006 (Jahresprävalenz)	*RZA*: 440 [399;481]/1 Mio. ≥ 50 J. *AAV*: 149 [126;174]/1 Mio. GPA: 98 [79;117], MPA: 28 [18;117], EGPA: 24 [14;35]	RZA: 612/219 AAV: 271/328 Pro 1 Mio. ≥ 50 J	Keine Standardisierung auf Standardpopulation
Hellmich 2021 [21]	AAV	Bundesweite GKV-Routinedaten (InGef)	Ca. 3 Mio. Versicherte ≥ 18 Jahre	M31.3 (GPA), M31.7 (MPA)	2013–2016 (kumuliert)	AAV: 256 ± 11/1 Mio. GPA: 210 ± 7/1 Mio. MPA: 46 ± 4/1 Mio.	–	Datenbank als repräsentativ für deutsche Bevölkerung nach Geschlecht und Alter angenommen, deshalb keine gesonderte Standardisierung
Thomschke 2018 [22]	JIA	Bundesweite GKV-Routinedaten	Ca. 12 Mio. Versicherte 0 bis 19 Jahre	M08.0–M09.0 (L40.5)	2009–2015 (jährliche Prävalenz im jeweiligen Jahresquerschnitt)	2009: 73,4/100.000 bis 2015: 101,5/100.000	119,8/58,9 je 100.000 (Durchschnittliche jährliche Prävalenz)	Keine Standardisierung
Luque Ramos 2017 [23]	JIA	Bundesweite BARMER-Routinedaten	238.000 Versicherte 16 bis 18 Jahre	M08. ×, M09.0	2008–2010	2008: 0,11 % 2009, 2010: 0,13 %	–	Keine Standardisierung

*AAV* ANCA-assoziierte Vaskulitis, *AS* Ankylosierende Spondylitis, *GKV* Gesetzliche Krankenversicherung, *GPA* Granulomatose mit Polyangiitis, *JIA* Juvenile idiopathische Arthritis, *MPA* Mikroskopische Polyangiitis, *PMR* Polymyalgia rheumatica, *PsA* Psoriasis-Arthritis, *RA* Rheumatoide Arthritis, *RZA* Riesenzellarteriitis, *SLE* Systemischer Lupus erythematodes

### Bewertung des „risk of bias“

Das Risiko der Verzerrung ist in Tab. 2 aufgelistet. Alle Studien haben im Gesamturteil ein moderates bis hohes Risiko der Verzerrung. Bezüglich der externen Validität erfüllten einige Studien eine gute Repräsentativität für die nationale Bevölkerung und hatten hier ein niedriges Verzerrungsrisiko. Bei 2 regionalen Analysen wurde das Risiko als hoch eingestuft. Einen „response bias“ gibt es bei den Routinedaten nicht; dies traf nur auf die Surveys zu und war bei den betreffenden Studien durch eine geringe Rücklaufquote hoch. Bezüglich der internen Validität erfolgte bei allen Routinedatenanalysen keine direkte Erhebung, dies ist nur bei den Surveys gegeben. Die Falldefinition betreffend wurde bei den Routinedatenanalysen ein niedriges Risiko angenommen, wenn mehrere Falldefinitionen getestet bzw. zusätzliche Kriterien für einen Einschluss berücksichtigt wurden.

**Table 2:** 

*Externe Validität* 1 War die Zielpopulation der Studie ein gutes Abbild der nationalen Bevölkerung in Bezug auf die relevanten Variablen? 2 War der Stichprobenrahmen ein wahres oder genaues Abbild der Zielpopulation? 3 Wurde für die Auswahl der Stichprobe eine Form der Zufallsauswahl verwendet, ODER wurde eine Volkszählung durchgeführt? 4 War die Wahrscheinlichkeit einer Nonresponse-Verzerrung minimal? *Interne Validität* 5 Wurden die Daten direkt bei den Probanden erhoben (im Gegensatz zu einem Stellvertreter)? 6 Wurde in der Studie eine akzeptable Falldefinition verwendet? 7 War das Studieninstrument zur Messung des Parameters von Interesse valide und zuverlässig? 8 Wurde für alle Probanden die gleiche Art der Datenerhebung verwendet? 9 War die Länge des kürzesten Prävalenzzeitraums für den Parameter von Interesse angemessen? 10 Waren der/die Zähler und der/die Nenner für den Parameter von Interesse angemessen? 11 Zusammenfassendes Item zum Gesamtrisiko der Studienverzerrung											
Referenz	1	2	3	4	5	6	7	8	9	10	11 (Gesamturteil)
Schmidt [4]	Niedrig	Niedrig	Niedrig	Hoch	Niedrig	Niedrig	Hoch	Niedrig	Niedrig	Niedrig	Moderat
Kienitz [5]	Niedrig	Niedrig	Niedrig	Niedrig	n.z.	Niedrig	Hoch	Niedrig	Niedrig	Niedrig	Moderat
Grellmann [6]	Niedrig	Niedrig	Niedrig	Niedrig	n.z.	Hoch	Hoch	Niedrig	Niedrig	Niedrig	Moderat
Strahl [7]	Hoch	Hoch	Niedrig	Niedrig	n.z.	Niedrig	Hoch	Niedrig	Niedrig	Niedrig	Hoch
Steffen [8]	Niedrig	Niedrig	Niedrig	Niedrig	n.z.	Niedrig	Hoch	Niedrig	Niedrig	Niedrig	Moderat
Hense [9]	Hoch	Niedrig	Niedrig	Niedrig	n.z.	Niedrig	Hoch	Niedrig	Niedrig	Niedrig	Moderat
Krüger [10]	Niedrig	Niedrig	Niedrig	Niedrig	n.z.	Niedrig	Hoch	Niedrig	Niedrig	Niedrig	Moderat
Deike [11]	Niedrig	Niedrig	Niedrig	Niedrig	n.z.	Hoch	Hoch	Niedrig	Niedrig	Niedrig	Moderat
Sewerin [12]	Niedrig	Niedrig	Niedrig	Niedrig	n.z.	Hoch	Hoch	Niedrig	Niedrig	Niedrig	Moderat
Reinhardt [13]	Hoch	Hoch	Niedrig	Niedrig	n.z.	Hoch	Hoch	Niedrig	Hoch	Hoch	Hoch
Sondermann [14]	Hoch	Hoch	Niedrig	Niedrig	n.z.	Hoch	Hoch	Niedrig	Niedrig	Niedrig	Hoch
Rech [15]	Niedrig	Niedrig	Niedrig	Niedrig	n.z.	Hoch	Hoch	Niedrig	Niedrig	Niedrig	Moderat
Schwarting [16]	Hoch	Niedrig	Niedrig	Niedrig	n.z.	Niedrig	Hoch	Niedrig	Niedrig	Niedrig	Moderat
Brinks [17]	Niedrig	Niedrig	Niedrig	Niedrig	n.z.	Hoch	Hoch	Niedrig	Niedrig	Niedrig	Moderat
Albrecht [18]	Hoch	Niedrig	Niedrig	Niedrig	n.z.	Hoch	Hoch	Niedrig	Niedrig	Niedrig	Hoch
Colombo [19]	Hoch	Niedrig	Niedrig	Niedrig	n.z.	Hoch	Hoch	Niedrig	Niedrig	Niedrig	Hoch
Herlyn [20]	Hoch	Hoch	Hoch	Hoch	Niedrig	Niedrig	Niedrig	Hoch	Niedrig	Niedrig	Hoch
Hellmich [21]	Niedrig	Niedrig	Niedrig	Niedrig	n.z.	Niedrig	Hoch	Niedrig	Niedrig	Niedrig	Moderat
Thomschke [22]	Niedrig	Niedrig	Niedrig	Niedrig	n.z.	Hoch	Hoch	Niedrig	Niedrig	Niedrig	Moderat
Luque Ramos [23]	Hoch	Niedrig	Niedrig	Niedrig	n.z.	Hoch	Hoch	Niedrig	Niedrig	Niedrig	Hoch

Zweistufige Beurteilung der einzelnen Kriterien: niedrig oder hoch, dreistufiges Gesamturteil: niedrig, moderat oder hoch

*n.z.* nicht zutreffend

### Studienergebnisse zur Prävalenz und Einordnung

#### Rheumatoide Arthritis

**Figure 1:**
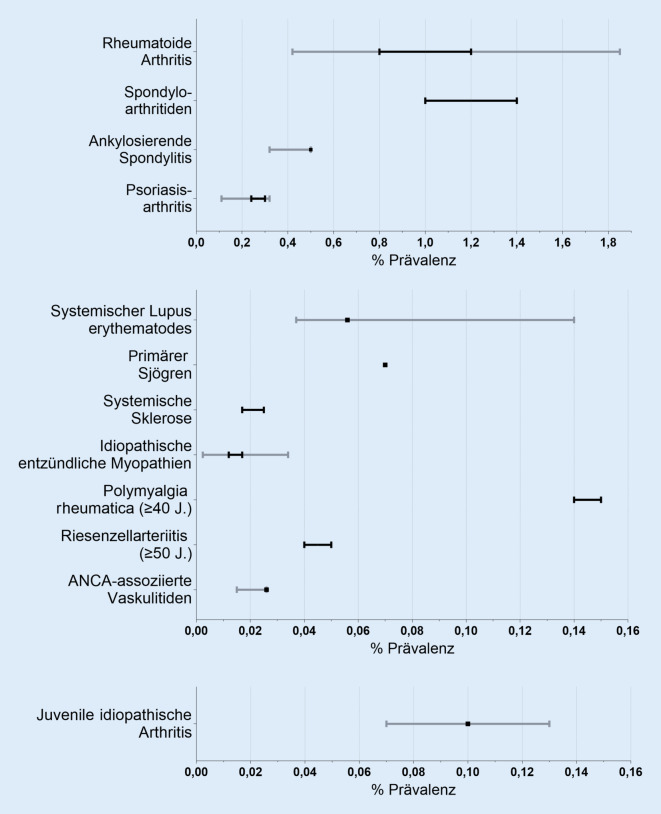

Für die rheumatoide Arthritis (RA) liegen 5 Routinedatenanalysen und patientenberichtete Angaben aus der NAKO-Gesundheitsstudie vor. Die Prävalenzangaben variieren je nach Falldefinition der RA von 0,4 % (Grellmann et al. [6]) bis 1,85 % (selbstberichtete ärztliche Diagnose aus der NAKO-Gesundheitsstudie [4]), s. Abb. 1. Die Prävalenz bei Grellmann et al. ist niedriger als in den anderen Routinedatenanalysen, weil nur die spezifischen ICD-10-Codes für RA M05.8, M06.0 und M06.8 eingeschlossen wurden. In der Versorgungsrealität werden aber am häufigsten die unspezifischen RA ICD-10-Codes M06.9 und M05.9 verwendet [24], sodass bei dieser Arbeit von einer Untererfassung von RA-Fällen ausgegangen werden muss. Bei den Selbstangaben der Befragten aus der NAKO-Studie ist eine Übererfassung durch häufige Verwendung des Begriffs „Rheuma“ auch bei Fingerpolyarthrose oder Gicht möglich; hier ist die Abgrenzung im Rahmen einer Befragung schwierig, obgleich explizit nach rheumatoider Arthritis bzw. Polyarthritis gefragt wurde. Auch bei der ausschließlichen Verwendung der ICD-10-Codes M05 und M06 gehen wir von einer Übererfassung aus, da v. a. die M06.9 häufig codiert wird, wenn (irgend)eine entzündlich rheumatische Erkrankung vorliegt. Mehrfachkodierungen, z. B. RA (M06) und Psoriasisarthritis sind häufig, und oftmals wird, wenn sich der Verdacht auf eine RA nicht bestätigt, der M06-Code nicht wieder gelöscht (Einschätzung aus der Praxis). Am plausibelsten erscheinen uns die Prävalenzschätzungen, bei denen zusätzlich zur ICD-Kodierung eine spezifische Medikation, eine Laboruntersuchung von Entzündungsmarkern oder eine fachärztliche Diagnose vorausgesetzt wurde [5, 7–9]. Basierend auf dieser Art der Falldefinition, schätzen wir, dass die Prävalenz der RA in der erwachsenen Bevölkerung in einem Bereich zwischen 0,8 % und 1,2 % liegt [25]. Das entspricht bei einer Bevölkerungszahl von 69,4 Mio. Erwachsenen im Jahr 2021 [26] derzeit ca. 560.000 bis 830.000 Betroffenen (Tab. 3).

**Table 3:** 

	Prävalenzangaben (in %) aus den Studien	Prävalenzannahme (in %) nach Analyse der Studien	Geschätzte Anzahl Betroffener^a^	Genauigkeit der Schätzung aus Sicht der Autor:innen
Rheumatoide Arthritis	0,42–1,85	0,8–1,2	560.000–830.000	Moderat
Spondyloarthritiden	1,0–1,4^f^	1,0–1,4	690.000–970.000	Niedrig
Ankylosierende Spondylitis	0,32–0,5	0,5	350.000	Niedrig
Psoriasisarthritis	0,11–0,32	0,24–0,32	170.000–220.000	Moderat
Systemischer Lupus erythematodes	0,037–0,14	0,056	39.000	Moderat
Sjögren (Sicca-Syndrom) davon primärer Sjögren	0,07–0,77	0,4–0,7 0,07	280.000–490.000 49.000	Niedrig
Systemische Sklerose	0,017–0,025^f^	0,017–0,025	12.000–17.000	Niedrig
Idiopathische entzündliche Myopathien	0,0024–0,034^f^	0,012–0,017 (Erwachsene + Kinder)	10.000–14.000^b^	Niedrig
Kollagenosen gesamt^g^	–	0,16–0,17	111.000–118.000	Niedrig
Polymyalgia rheumatica	0,14–0,15 (≥ 40 Jahre)	0,14–0,15 (≥ 40 Jahre)	66.000–71.000^c^	Niedrig
Riesenzellarteriitis	0,04–0,05 (≥ 50 Jahre)	0,04–0,05 (≥ 50 Jahre)	15.000–19.000^d^	Niedrig
ANCA-assoziierte Vaskulitiden	0,015–0,026	0,026	18.000	Moderat
Entzündlich rheumatische Erkrankungen bei Erwachsenen	–	2,2–3,0	Ca. 1,5–2,1 Mio. Erwachsene	Moderat
Juvenile idiopathische Arthritis	0,07–0,13	0,10	Ca. 14.000 Kinder und Jugendliche^e^	Moderat

^a^Bezogen auf 69,4 Mio. Erwachsene

^b^83,2 Mio. Erwachsene und Kinder und Jugendliche

^c^47,5 Mio. Erwachsene ≥ 40 Jahren

^d^37,5 Mio. Erwachsene ≥ 50 Jahren

^e^13,9 Mio. Kinder und Jugendliche < 18 Jahren in der deutschen Bevölkerung im Jahr 2021 [26]

^f^Internationale bzw. ältere Daten aus Deutschland

^g^Systemischer Lupus erythematodes, primärer Sjögren, systemische Sklerose und Myopathien

#### Axiale Spondyloarthritis

Über die Häufigkeit axialer Spondyloarthritiden (axSpA) gibt es wenig Daten aus Deutschland. In der NAKO-Gesundheitsstudie gaben 0,49 % einen Morbus Bechterew/ankylosierende Spondylitis an [4]. Die aus der NAKO-Studie berichteten Häufigkeiten können aber nicht als exakte Prävalenzschätzungen angesehen werden. Die Antwortrate im Survey war mit 18 % sehr niedrig, und die Kohorte umfasste nur Personen im Alter von 20 bis 69 Jahren, sodass ein Selektionsbias wahrscheinlich ist. Eine Routinedatenanalyse mit der Datenbank des Instituts für angewandte Gesundheitsforschung (InGef) schätzt eine Prävalenz der ICD-10-M45-Diagnose von 0,32 % im Jahr 2013, anhand dieser die Anzahl an Erwachsenen mit einer AS in Deutschland auf 217.400 hochgerechnet wurde [10]. Vergleichbar war der Anteil von 0,31 % mit einer M45-Diagnose im Jahr 2020 in einer BARMER-Routinedatenanalyse, die aber nicht primär auf eine Prävalenzschätzung ausgerichtet war [27]. Ob die M45-Diagnose tatsächlich alle Personen mit AS erfasst, bleibt unsicher. Im norwegischen Patientenregister lag die Prävalenz der axSpA im Jahr 2017 bei 0,41 % in der erwachsenen Bevölkerung [28]. Zur axSpA zählen über die ankylosierende Spondylitis (AS) hinaus auch nichtradiologische Formen, was die Anzahl Betroffener deutlich erhöht. Hierzu gibt es keine aktuelle Studie aus Deutschland. Basierend auf älteren Daten aus der 1998er Berlin-Studie wurde die Prävalenz der AS in Deutschland bisher auf 0,5 % und die der SpA insgesamt auf 1–1,4 % geschätzt [29]. Dies stimmt gut mit bisherigen Prävalenzschätzungen aus den USA (AS 0,52–0,55 %, axSpA 1–1,4 %) überein [30] und entspräche bezogen auf Deutschland einer Anzahl von ca. 350.000 Personen mit einer AS und insgesamt ca. 690.000 bis 970.000 Personen mit einer SpA.

#### Psoriasisarthritis

Fünf Routinedatenanalysen liegen für die Prävalenzschätzung der Psoriasisarthritis (PsA) vor. Bei Grellmann et al. [6] lag die jährliche Prävalenz für die Jahre 2012 bis 2016 zwischen 0,27 % und 0,32 %, wobei als Diagnosekriterium die ICD-10-Codes M07.0–3, L40.5 verwendet wurden. Vergleichbar ist die Prävalenzangabe von Reinhardt et al. mit 0,29 % im Jahr 2010 und vergleichbaren Diagnosekriterien [13]. Sewerin et al. schätzen anhand der Diagnoseprävalenzen von 0,21 % (Männer) und 0,25 % (Frauen) aus dem Jahr 2012, dass es im Jahr 2018 mindestens 200.000 Betroffene in Deutschland gab [12]. Bei gleicher Datengrundlage wurde von Deike et al. die Prävalenz für 2012 mit 0,24 % berichtet [11]. Mit Daten aus dem InGef identifizierten Rech et al. im Jahr 2012 4390 Personen mit einer PsA in einem Kollektiv von 2,8 Mio. Versicherten. Dies entspricht einer Prävalenz von 0,15 %, wobei Rech et al. den Code M07.2 (Spondylitis psoriatica) nicht eingeschlossen haben [15]. Ebenso scheint die Prävalenzangabe von Sondermann et al. mit 0,11 % eine Unterschätzung zu sein, da hier nur der Code L40.5 in 2 Quartalen aus dem Jahr 2014 berücksichtigt wurde [14]. Zieht man internationale Daten hinzu, lag die gepoolte Prävalenz der PsA in einer Metaanalyse mit Einschluss von 28 Studien bei 0,13 % (95 %-Konfidenzintervall: 0,11–0,16 %) [31] – mit großer Variabilität der einzelnen Studien und deutlich niedriger als die Prävalenz aus dem norwegischen Patientenregister von 0,46 % in der erwachsenen Bevölkerung [31]. Zwei Studien mit Psoriasiskollektiven aus Deutschland zeigten nach klinischer Untersuchung, dass 20 % der Psoriasispatient:innen auch eine PsA hatten; viele davon nicht diagnostiziert [32, 33]. In einer weiteren Studie von 2014 lag der Anteil an rheumatologisch bereits diagnostizierter PsA im untersuchten Psoriasiskollektiv bereits bei 19 %, und weitere 11 % wurden durch rheumatologische Nachuntersuchung neu diagnostiziert [34]. Es ist daher anzunehmen, dass der Anteil an PsA bei Psoriasis deutlich höher liegen könnte als bisher beschrieben [1]. Da undiagnostizierte Fälle in Routinedaten der Krankenkassen nicht berücksichtigt werden und der Diagnosecode spezifischer ist als der M06-RA-Code, gehen wir bei den PsA-Routinedaten eher von einer Unterschätzung der Fallzahlen aus. Daher schätzen wir für Deutschland eine Prävalenz von 0,24–0,32 % für die PsA, welches ca. 170.000 bis 220.000 Betroffenen entspräche.

### Kollagenosen

#### Systemischer Lupus erythematodes

Für den systemischen Lupus erythematodes (SLE) wurden 2 Routinedatenanalysen und Daten aus der NAKO-Gesundheitsstudie eingeschlossen. Anhand der Prävalenz der SLE-Diagnose im Jahr 2002 von 36,7 (34,3–39,3)/100.000 haben Brinks et al. die Zahl an Personen mit einem SLE auf 31.000 Betroffene in 2010 und einem weiteren Anstieg bis 2020 hochgerechnet [17]. Bei Schwarting et al. lag die Prävalenz für 2014 mit 55,8/100.000 noch höher [16], obwohl in dieser Arbeit bei ambulanter ICD-Diagnose zusätzlich eine SLE-spezifische Diagnostik, Medikation oder Facharztdiagnose vorausgesetzt wurde. International gibt es eine große Variabilität mit insgesamt niedrigeren Prävalenzangaben in Europa im Vergleich zu den USA [35]. Die geschätzte Prävalenz aus UK lag mit 97/100.000 in 2012 deutlich höher als die aus Deutschland und verzeichnete einen Anstieg im Vergleich zu den Jahren vor 2012 [36]. In der NAKO-Gesundheitsstudie gaben 0,14 % der Befragten einen jemals ärztlich diagnostizierten SLE an [4]. Hier bleibt eine Unsicherheit, ob auch kutane Formen von den Befragten als SLE angekreuzt wurden und diese Anzahl deshalb als Überschätzung eingestuft werden sollte. Ordnet man die Ergebnisse in die internationalen Angaben ein, erscheint eine Prävalenz von ca. 0,056 % plausibel, welches in etwa 39.000 Betroffenen entspräche.

#### Sjögren

Eine Routinedatenanalyse zeigt eine hohe Prävalenz der Abrechnungsdiagnose des Sjögren-Syndroms (M35.0: Sicca-Syndrom [Sjögren-Syndrom]) zwischen 0,68 % und 0,77 % in den Jahren 2007 bis 2018 [18]. Hierbei muss berücksichtigt werden, dass die Abrechnungsdiagnose nicht zwischen primären und sekundären Formen unterscheidet. Die Einordnung im ICD-10 erfolgt unter „Sonstige Krankheiten mit Systembeteiligung des Bindegewebes“, sodass hierunter eigentlich nur primäre und sekundäre Formen bei Kollagenosen kodiert werden und nicht solche, die bei anderen Erkrankungen auftreten. Die Alters- und Geschlechterverteilung des in den Routinedaten kodierten Sjögren entspricht aber nicht der anhand der klinischen Erfahrung erwarteten Verteilung. Daher vermuten wir hier eine deutliche Überschätzung der realen Prävalenz bei überhäufiger Kodierung aufgrund einer Sicca-Symptomatik. Für Deutschland wurde unter Einbeziehung sekundärer Formen bisher eine Prävalenz von mindestens 0,4 % angenommen [37]. In der NAKO-Gesundheitsstudie gaben 0,07 % der Befragten ein Sjögren-Syndrom an [4], was eher dem primären Sjögren entsprechen könnte, da nicht explizit nach primären oder sekundären Formen gefragt wurde. Die globale Prävalenz des primären Sjögren wird mit 60,8 (95 % Konfidenzintervall (KI) 43,7–77,9)/100.000 Einwohner angegeben; mit höheren Prävalenzen in Europa [38]. Daher schätzen wir den Anteil an Personen mit einem primären Sjögren auf 0,07 % und den Anteil inklusive sekundärer Formen auf 0,4–0,7 % – allerdings mit großer Unsicherheit bezüglich der Abgrenzung zur Sicca-Symptomatik bei nichtentzündlichen Erkrankungen. Dies entspräche ca. 49.000 (primäres Sjögren) bzw. 280.000 bis 490.000 (primär und sekundär) Betroffenen.

Zur systemischen Sklerose und zu idiopathischen entzündlichen Myopathien liegen aus Deutschland keine Studien vor, wir berichten daher internationale Angaben.

#### Systemische Sklerose

Zur systemischen Sklerose gibt es relativ konsistente Prävalenzangaben aus Schweden aus dem Jahr 2015 von 22,7/100.000 [39], aus Dänemark mit 17,9 bis 19,2/100.000 in den Jahren 2009 bis 2016 [40] und aus Großbritannien von 17,1 bis 25,4/100.000 in den Jahren 2000 bis 2012 [41]. Diese decken sich gut mit unpublizierten Daten aus dem Deutschen Netzwerk für Systemische Sklerodermie (DNSS), die auf eine ungefähre Prävalenz von 20/100.000 schließen lassen (Mitteilung Prof. Blank, Heidelberg). Wir legen daher einen Bereich von 17 bis 25/100.00 für eine Schätzung zugrunde, das entspräche ca. 12.000 bis 17.000 Erwachsenen.

#### Idiopathische entzündliche Myopathien

Die Prävalenz der idiopathischen entzündlichen Myopathien lag im schwedischen Patientenregister im Jahr 2012 bei 14 (95 % CI 13–15)/100.000. Hierbei wurden Dermatomyositis, Polymyositis, Einschlusskörpermyositis, juvenile Dermatomyositis und unspezifische Myositis berücksichtigt. Wenn eine breitere oder striktere Falldefinition gewählt wurde, lag die Prävalenz bei 12 (95 % KI 11–13) bis 17 (95 % KI 16–18)/100.000 [42]. Internationale Prävalenzen aus einem systematischen Review liegen in einem Bereich von 2,4 bis 33,8/100.000 [43]. Bei fehlenden nationalen Daten legen wir den Bereich von 12 bis 17/100.000 aus der schwedischen Studie für eine Schätzung zugrunde, das entspräche bei einer Bevölkerungszahl von 83,2 Mio. ca. 10.000 bis 14.000 Betroffenen (inklusive Kindern und Jugendlichen mit einer juvenilen Myositis).

Addiert man die Prävalenzangaben der Kollagenosen auf (SLE, primäres Sjögren und internationale Angaben zur systemischen Sklerose und zu Myositiden), ergibt sich eine ungefähre Prävalenz von 0,16–0,17 %, was ca. 111.000 bis 118.000 Betroffenen entspräche.

#### Polymyalgia rheumatica

Zur Polymyalgia rheumatica (PMR) liegt lediglich eine Analyse mit Daten der AOK Württemberg vor, die für das Jahr 2019 eine alters- und geschlechtsstandardisierte Prävalenz der PMR von 145 (95 % KI: 143–147)/100.000 bei Personen im Alter ab 40 Jahren berechnet hat [19]. Die Prävalenz wurde nicht für über 50-Jährige berechnet, sodass wir anhand dieser Daten keine Schätzung für die klassifikationsgemäße Altersgruppe ab 50 Jahren bestimmen können. Internationale Angaben liegen mit Prävalenzschätzungen von 370 bis 850/100.000 bei Personen ab 50 Jahren deutlich höher [19, 44]. Legen wir die Zahlen aus Baden-Württemberg zugrunde, entspräche dies bei 47,5 Mio. Erwachsenen ≥ 40 Jahren im Jahr 2021 [26] ca. 66.000 bis 71.000 Betroffenen.

#### Riesenzellarteriitis

Die Prävalenz der Riesenzellarteriitis (RZA) wurde in einem regionalen Survey in Lübeck und Bad Segeberg 1994 und 2006 untersucht. Im Jahr 1994 lag die Prävalenz bei 24 (95 % KI:14–35)/100.000 und im Jahr 2006 bei 44 (95 % KI: 40–48)/100.000 für Personen ab 50 Jahre [20]. Im internationalen Kontext liegen diese Zahlen eher im unteren Bereich: Ein internationaler Systematic Review berichtete sehr heterogene Prävalenzen von 20 (95 % KI: 16–24)/100.000 (Türkei) bis 250 (95 % KI: 110–390)/100.000 (UK), jeweils bezogen auf Erwachsene ab 50 Jahre [44]. Rechnen wir die Daten aus Schleswig-Holstein hoch, entspräche dies bei 37,5 Mio. Erwachsenen ≥ 50 Jahren ca. 15.000 bis 19.000 Betroffenen.

#### ANCA-assoziierte Vaskulitiden

Für die ANCA-assoziierten Vaskulitiden (AAV) zeigte die regionale Abfrage von Herlyn et al. ebenso wie bei der RZA eine Verdopplung der Prävalenz von 74/1 Mio. im Jahr 1994 auf 149 (95 % KI:126–174)/1 Mio. im Jahr 2006 [20]. Routinedaten aus dem InGef aus den Jahren 2013 bis 2016 ergaben anhand der ICD-Diagnosecodes eine Prävalenz für die granulomatöse Polyangiitis (GPA) von 210/1 Mio. und für die mikroskopische Polyangiitis (MPA) von 46/1 Mio., entsprechend für die AAV insgesamt eine Prävalenz von 256/1 Mio. Anhand dieser Daten schätzen Hellmich et al., dass ca. 17.500 Menschen mit einer AAV (GPA und MPA) in Deutschland leben [21]. Eine aktuelle Metaanalyse ergab für alle internationalen Studien eine gepoolte AAV Prävalenz von 198 (95 % KI:187–210)/1 Mio. mit sehr heterogenen Prävalenzen in den einzelnen Studien (44,8 bis 421/1 Mio.). Auch in anderen Studien zeigte sich ein Anstieg der AAV-Prävalenz über die Jahre [45]. Rechnen wir den Anstieg des Lübecker Surveys von 2006 an weiter fort, erscheint die Prävalenzschätzung der AAV von Hellmich et al. mit den Daten aus Norddeutschland konform. Wir schätzen daher anhand der Bevölkerungszahl von 2021 eine Zahl von etwa 18.000 Betroffenen.

#### Schätzung der Gesamtzahl an Personen mit einer entzündlich rheumatischen Erkrankung

**Figure 2:**
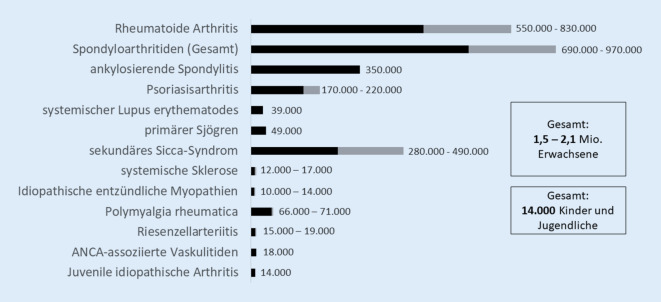

Im Jahr 2016 haben wir geschätzt, dass etwa 2 % der erwachsenen Bevölkerung von einer entzündlich rheumatischen Krankheit betroffen sind, welches einer Anzahl von ca. 1,45 Mio. Betroffenen entsprach [1]. Die Prävalenzschätzungen aus der Literatur sind seitdem krankheitsübergreifend angestiegen, was bei einer höheren Lebenserwartung, gesunkener Mortalität und verbesserter Frühdiagnostik plausibel erscheint. Seit 2014 (Daten unserer letzten Schätzung) ist der Anteil der über 80-Jährigen um 23 % von 5,6 % auf 7,3 % der Bevölkerung in Deutschland angestiegen. Bei den 60- bis 80-Jährigen gab es einen Anstieg um 4,4 % auf insgesamt 22 % der Bevölkerung [26], welches sicher mit einem Anstieg der Prävalenz chronisch entzündlich rheumatischer Erkrankungen einhergeht. Wir schätzen daher heute anhand der zur Verfügung stehenden Literatur, dass etwa 2,2–3,0 % der erwachsenen Bevölkerung von einer entzündlich rheumatischen Erkrankung betroffen sind, entsprechend ca. 1,5 bis 2,1 Mio. Erwachsenen (Abb. 2).

#### Entzündlich rheumatische Erkrankungen bei Kindern und Jugendlichen

Für Kinder und Jugendliche gibt es Daten zur juvenilen idiopathischen Arthritis (JIA) aus dem Versorgungsatlas. Hier wiesen im Jahr 2009 73,4/100.000 und im Jahr 2015 101,5/100.000 der Kinder und Jugendlichen eine JIA-Diagnose auf [22]. Bei den Jugendlichen zwischen 16 und 18 Jahren lag die Prävalenz der JIA-Diagnose in Routinedaten der BARMER zwischen 0,11 % in 2008 und 0,13 % in 2009 und 2010 (kumuliert) [23]. Daten aus UK aus dem Jahr 2018 zeigen einen deutlichen Unterschied der Prävalenzrate bei Anwendung der JIA-Diagnosecodes (56,3 [95 % KI 53,2–59,6]/100.000) und bei klinisch validierten Fällen (30,6 [95 % KI 27,9–33,4]/100.000, altersstandardisiert 43,5/100.000) [46]. Die Abweichungen der Prävalenzschätzungen von Costello et al. verdeutlichen noch einmal die Unsicherheit, die die Auswertung von Routinedaten für Prävalenzschätzungen mit sich bringt. Gleichbleibende Inzidenzraten der JIA-Diagnose in Deutschland [22] und auch in Dänemark [47] sprechen dafür, dass der Anteil an Kindern und Jugendlichen mit einer JIA weitgehend stabil ist. Für die JIA bleiben wir bei der Schätzung von 2016, dass ca. 1 von 1000 Kindern von einer JIA betroffen ist. Das entspricht bei 13,9 Mio. Kindern und Jugendlichen < 18 Jahren in der deutschen Bevölkerung im Jahr 2021 [26] ca. 14.000 Kindern und Jugendlichen. Für den juvenilen SLE, die juvenilen Myositiden und Vaskulitiden gibt es keine Daten aus Deutschland. Für die Untergruppe der juvenilen PsA lag die Prävalenz in der Arbeit von Reinhardt et al. bei 0,01 % [13].

## Fazit für die Praxis

- Die systematische Literaturrecherche zur Prävalenz ERE in Deutschland zeigt in vielen Studien einen Anstieg der Prävalenzen im Vergleich zu früheren Auswertungen. Fast alle Studien basieren auf Routinedaten, und alle Studien haben ein moderates bis hohes Verzerrungsrisiko. In Routinedaten werden nur Diagnosen und nicht der bestehende Krankheitsstatus dokumentiert, darüber hinaus erschweren fehlerhafte und mehrfache/überlappende Kodierungen eine zuverlässige Bestimmung der Prävalenz. Da es keine mehrstufigen Bevölkerungsstudien gibt, sind die vorliegenden Daten die einzigen verfügbaren, aber unsicheren Quellen für Prävalenzschätzungen. Anhand dieser Daten schätzen wir, dass heute etwa 2,2–3 % der Erwachsenen in Deutschland eine entzündlich rheumatische Erkrankung und 0,1 % der Kinder und Jugendlichen eine juvenile Arthritis haben, was einer Zahl von 1,5 bis 2,1 Mio. Erwachsenen bzw. ca. 14.000 Kindern und Jugendlichen entspricht.

### Supplementary Information




