# Supplementary material for: Systematic review to estimate the prevalence of inflammatory rheumatic diseases in Germany. German version
Source: Z Rheumatol. 2023 Jan 2;82(9):727–38. [Article in German] doi: 10.1007/s00393-022-01305-2 (PMC10627889; doi:10.1007/s00393-022-01305-2)

Suchstrategie zur systematischen Literatursuche zur Prävalenz entzündlich-rheumatischer Erkrankungen in Deutschland

PubMed/Medline:

("prevalence"[MeSH Terms] OR "prevalence"[Title/Abstract] OR "frequencies"[Title/Abstract]) AND ("rheumatic disease*"[Title/abstract] OR "rheumatoid arthritis"[Title/Abstract] OR “ankylosing spondylitis” [Title/abstract] OR “spondyloarthritis” [Title/abstract] OR “juvenile arthritis” [title/abstract] OR "systemic lupus erythematosus” [Title/abstract] OR "polymyalgia rheumatica"[Title/abstract] OR "psoriatic arthritis"[Title/abstract] OR "myositis"[Title/abstract]) OR "giant cell arteritis"[Title/abstract] OR "sjogren*"[Title/abstract] OR "ANCA-associated vasculitis"[Title/abstract]) AND ("german"[Title/Abstract] OR "Germany"[Title/Abstract]) AND 2014/01/01:2022/10/31[Date - Publication]

Flow chart

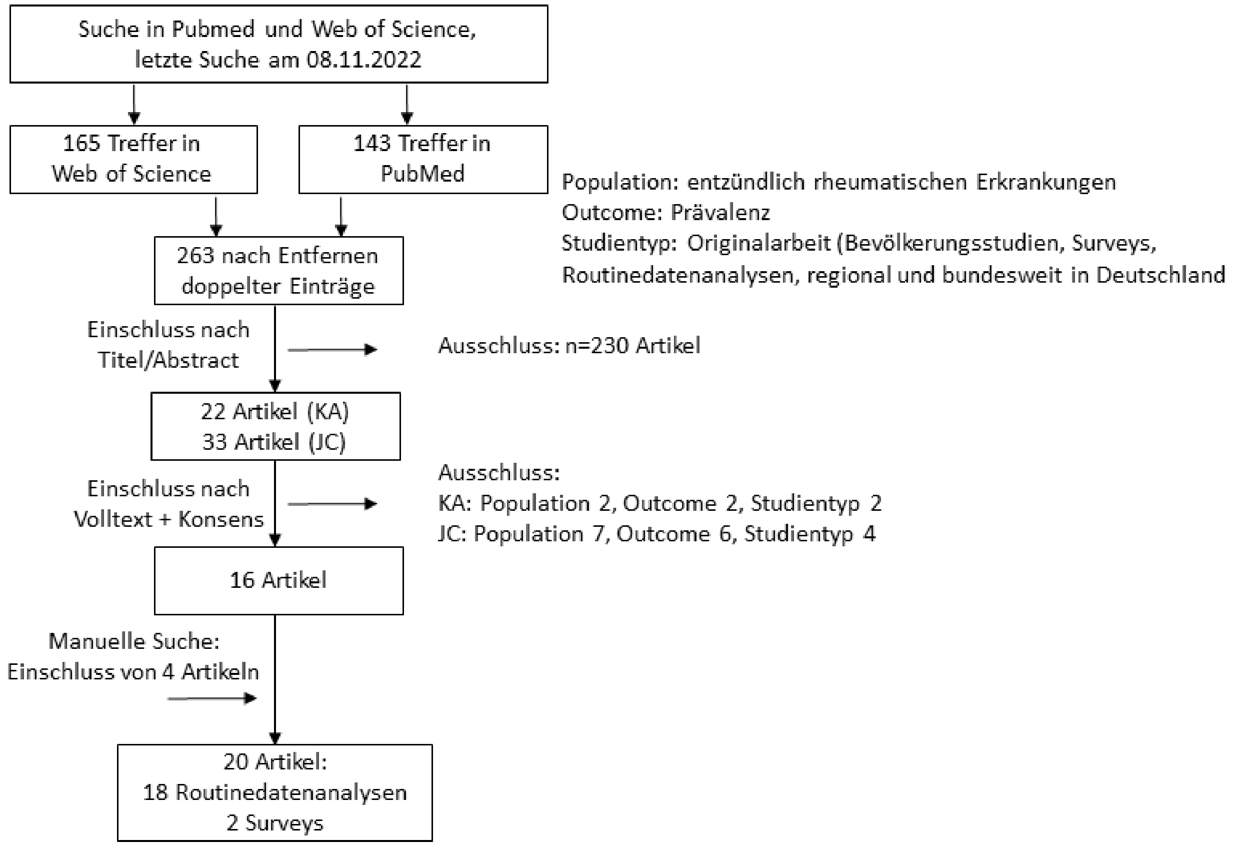

Supplement: Supplementary file 1 [file 393_2022_1305_MOESM1_ESM.pdf]
